# Supplementary material for: Cloning and expression analysis of cinnamoyl-CoA reductase (CCR) genes in sorghum
Source: PeerJ. 2016 May 19;4:e2005. doi: 10.7717/peerj.2005 (PMC4878380; doi:10.7717/peerj.2005)
Supplement: Supplemental Information 1 [file peerj-04-2005-s001.docx]

SbCCR1 sequencing result (only CDS)

ATGACCGTCGTCGACGCCGTCTCCACTGATGCCGCCGGCGCCGCCCCAGCTGCCGCCGCGGCGCCGGTGGTGGTGGCGCAGCCCGGGAACGGGCAGACCGTGTGCGTCACCGGCGCGGCCGGGTACATCGCCTCGTGGCTCGTCAAGATGCTGCTCGAGAAGGGATACACTGTCAAGGGCACCGTCAGGAACCCAGATGACCCGAAGAACGCGCACCTCAAGGCGCTGGACGGCGCGGCCGAGCGGCTGATCCTCTGCAAGGCCGACCTCCTGGACTACGACGCCATCTGCCGCGCCGTGCAGGGCTGCCAGGGCGTCTTCCACACCGCCTCCCCCGTCACCGACGATCCGGAGCAAATGGTGGAGCCGGCGGTGCGCGGCACGGAGTACGTGATCAACGCGGCGGCGGAGGCCGGCACGGTGCGGCGGGTGGTGTTCACGTCCTCCATCGGCGCGGTGACCATGGACCCCAGCCGCGGGCCCGACGTCGTGGTCGACGAGTCGTGCTGGAGCGACCTCGAGTTCTGCAAGAAAACCAGGAACTGGTACTGCTACGGCAAGGCGGTGGCGGAGCAGGCGGCGTGGGACGCGGCCCGGCAGCGCGGCGTGGACCTGGTGGTGGTGAACCCGGTGCTGGTGGTGGGCCCGCTGCTGCAGCCGACGGTGAACGCCAGCATCGCGCACGTGCTCAAGTACCTGGACGGCTCCGCGCGCACCTTCGCCAACGCCGTGCAGGCGTACGTGGACGTCCGCGACGTCGCCGACGCGCACCTCCGCGTCTTCGAGAGCCCCGCCGCGTCCGGCCGATACCTCTGCGCCGAGCGCGTCCTCCACCGCGAGGACGTCGTCCGCATCCTCGCCAAGCTCTTCCCCGAGTACCCCGTCCCCACCAGGTGCTCCGACGAGGTGAACCCGCGGAAGCAGCCGTACAAGTTCTCGAACCAGAAGCTCCGGGACCTGGGATTGGAGTTCCGGCCGGTGAGCCAGTCGCTCTACGATACGGTGAAGAACCTTCAGGAGAAGGGCCACCTGCCGGTGCTCGGAGAGCAGACGACGGAGGCCGACAAGGAGGAGGCCAACGCCGCCGCCGAGGTGCAGCAGGGAGGAATCGCCATCCGTGCG

Protein sequence

MTVVDAVSTDAAGAAPAAAAAPVVVAQPGNGQTVCVTGAAGYIASWLVKMLLEKGYTVKGTVRNPDDPKNAHLKALDGAAERLILCKADLLDYDAICRAVQGCQGVFHTASPVTDDPEQMVEPAVRGTEYVINAAAEAGTVRRVVFTSSIGAVTMDPSRGPDVVVDESCWSDLEFCKKTRNWYCYGKAVAEQAAWDAARQRGVDLVVVNPVLVVGPLLQPTVNASIAHVLKYLDGSARTFANAVQAYVDVRDVADAHLRVFESPAASGRYLCAERVLHREDVVRILAKLFPEYPVPTRCSDEVNPRKQPYKFSNQKLRDLGLEFRPVSQSLYDTVKNLQEKGHLPVLGEQTTEADKEEANAAAEVQQGGIAIRA

SbCCR2-1 sequencing result (only CDS)

ATGCCAACAGCAGAGACGACGACGCCCGTGCCGCCAGCGCTCTCCGGGCAAGGCCGGACAGTTTGCGTCACCGGAGCTGGAGGGTTCATCGCCTCCTGGCTTGTCAAGCGCCTCCTCGAGAAGGGTTACACAGTCCGTGGCACGGTCAGGAACCCTGTCGATCCAAAGAACGACCACCTGAGGGCCCTTGACGGCGCCGCCGATCGCCTCGTCCTCCTGCGTGCCGATCTGCTGGATCCAGAAAGCCTTGTCGAGGCCTTCTCCGGCTGCGACGGCGTCTTCCACGCCGCCTCCCCGGTCACCGATGACCCTGAGATGATGATCGAGCCAGCAATCCGGGGCACACAATATGTGATGACGGCGGCGGCAGACACCGGCGTCAAGCGCGTCGTGTTCACGTCCTCCATCGGCACAGTGTACATGAACCCCTACCGTGAACCCAACAAGCCTGTCGACGACACCTGCTGGAGCGATCTTGAGTATTGCAAGAATACACAGAACTGGTATTGCTACGCCAAGACAGTGGCGGAGCAGGGCGCATGGGAGGTGGCGCGGAAGCGAGGCCTGGACCTGATCGTGGTGAACCCGGTGCTGGTGCTGGGTCCGTTGCTGCAGCCAACAGTGAACGCCAGCACGGACCACGTGATGAAGTACCTGACGGGGTCGGCCAAGACGTACGTGAACGCCGCGCAGGCGTACGTGCACGTCCAGGACGTCGCGGAGGCGCACGTCCGGGTGTACGAGGCACCCTACGCGCATGGGCGCTACATCTGCGCCGAGAGCACCCTCCACCGCGGCGAGCTCTGCCGCATCCTCGCTAAGCTCTTCCCAGAGTACCCCATACCCACAAAGTGCAAGGACGACGTGAACCCTCCGGTGACAGGATACAAGTTCACGAACCAGCGGCTCAAGGATCTTGGGATGGACTTTGTGCCGGTGCTGCAGTGCCTCTACGAGACAGTGAAGAGCCTCCAGGAGAAAGGCATGCTGCCCGTGCTTCCGCCAAAAGACGACCAGGACCAACAACTCCACAAATCA

Protein sequence

MPTAETTTPVPPALSGQGRTVCVTGAGGFIASWLVKRLLEKGYTVRGTVRNPVDPKNDHLRALDGAADRLVLLRADLLDPESLVEAFSGCDGVFHAASPVTDDPEMMIEPAIRGTQYVMTAAADTGVKRVVFTSSIGTVYMNPYREPNKPVDDTCWSDLEYCKNTQNWYCYAKTVAEQGAWEVARKRGLDLIVVNPVLVLGPLLQPTVNASTDHVMKYLTGSAKTYVNAAQAYVHVQDVAEAHVRVYEAPYAHGRYICAESTLHRGELCRILAKLFPEYPIPTKCKDDVNPPVTGYKFTNQRLKDLGMDFVPVLQCLYETVKSLQEKGMLPVLPPKDDQDQQLHKS

Sb-CCR2-2 sequencing result (only CDS)

ATGGCCGTCGTCGTGTGCGTCACCGGCGCCGGCGGCTTCATCGGGTCGTGGATCGTTAAGATCCTCCTCGCCCGCGGGTACGCCGTCCGGGGCACCTCCCGCCGCGCAGATGACCCCAAGAACGCGCACCTGTGGGCGCTCGACGGCGCGGCGGAGCGCCTCACCATGCTGCAGGTGGACCTGCTCGACCGTGCCAGCCTCCGCGCCGCATTCCGCGGCTGCGACGGCGTCATCCACACCGCCTCGCCGATGCACGACAACCCCGAGGAGATCATCGAGCCGATTATCGTCGGGACGCGGAACGTCGTCGAGGCCGCGGCCGACGCCGGCGTGCGGCGCCTGGTGCTGTCCTCCACCATCGGCACCATGTACATGGATCCGCGCCGCGACCCGGACGCGGCACTCGGTGACTCCAGCTGGAGCGACCTCGAATACTGCAAGAGCACCAAGAACTGGTACTGCTACGCGAAGACGATCGCGGAGCAGGGCGCGTGGGAGGCGGCGCGGGCGCGGGGGCTGGACCTGGCGGTGGTCATCCCGGTGGTAGTGCTCGGCGAGCTGCTGCAGCCCAACATGAACACCAGCACCCTGCACATCCTCAAGTACCTCACTGGGCAGACCAAGGAGTACGTCAACGAATCGCATGCCTACGTGCACGTCAAGGACGCTGCCGAGGCGCACGTCAGGGTGCTCGAGGCGCCTGGCGCCGGCGGGCGGCGGTATGTCTGCGCTGAGCGCACTCTGCACCGCGGCGAGCTCTGCCGTATCCTCGTCGGACTCTTCCCGGAGTACCCTATTCCGACAAGGTGCAAGGATCAGGTGAATCCACCAAAGAAGGGTTACAAGTTTACAAACCAGCCTCTGAAGGACCTAGGAATCAAGTTCACGCCAGTGCATGAATACCTGTATGAAGCAGTGAAATCCCTGCAAGAAAAGGGATTCCTCCAGAAGACCTCTAACACCAAGGTGCCTGAACGACGCAGCCGCCTGCCTGAACAATCACAACCACCCGTATTGATTTCAAAACTT

Protein sequence

MAVVVCVTGAGGFIGSWIVKILLARGYAVRGTSRRADDPKNAHLWALDGAAERLTMLQVDLLDRASLRAAFRGCDGVIHTASPMHDNPEEIIEPIIVGTRNVVEAAADAGVRRLVLSSTIGTMYMDPRRDPDAALGDSSWSDLEYCKSTKNWYCYAKTIAEQGAWEAARARGLDLAVVIPVVVLGELLQPNMNTSTLHILKYLTGQTKEYVNESHAYVHVKDAAEAHVRVLEAPGAGGRRYVCAERTLHRGELCRILVGLFPEYPIPTRCKDQVNPPKKGYKFTNQPLKDLGIKFTPVHEYLYEAVKSLQEKGFLQKTSNTKVPERRSRLPEQSQPPVLISKL

zmCCR1

MTVVDAVVSSTDAGAPAAAATAVPAGNGQTVCVTGAAGYIASWLVKLLLEKGYTVKGTVRNPADDPKNAHLKALDGAAERLILCKADLLDYDAICRAVQGCQGVFHTASPVTDDPEQMVEPAVRGTEYVINAAAEAGTVRRVVFTSSIGAVTMDPKRGPDVVVDESCWSDLEFCEKTRNWYCYGKAVAEQAAWETARRRGVDLVVVNPVLVVGPLLQATVNASIAHILKYLDGSARTFANAVQAYVDVRDVADAHLRVFESPRASGRHLCAERVLHREDVVRILAKLFPEYPVPARCSDEVNPRKQPYKFSNQKLRDLGLQFRPVSQSLYDTVKNLQEKGHLPVLGERTTTEAADKDAPTAEMQQGGIAIRA

ATCCR1

MPVDVASPAGKTVCVTGAGGYIASWIVKILLERGYTVKGTVRNPDDPKNTHLRELEGGKERLILCKADLQDYEALKAAIDGCDGVFHTASPVTDDPEQMVEPAVNGAKFVINAAAEAKVKRVVITSSIGAVYMDPNRDPEAVVDESCWSDLDFCKNTKNWYCYGKMVAEQAAWETAKEKGVDLVVLNPVLVLGPPLQPTINASLYHVLKYLTGSAKTYANLTQAYVDVRDVALAHVLVYEAPSASGRYLLAESARHRGEVVEILAKLFPEYPLPTKCKDEKNPRAKPYKFTNQKIKDLGLEFTSTKQSLYDTHRKNPWKMALRSGLEKLINSLKYPP

ATCCR2

MLVDGKLVCVTGAGGYIASWIVKLLLERGYTVRGTVRNPTDPKNNHLRELQGAKERLTLHSADLLDYEALCATIDGCDGVFHTASPMTDDPETMLEPAVNGAKFVIDAAAKAKVKRVVFTSSIGAVYMNPNRDTQAIVDENCWSDLDFCKNTKNWYCYGKMLAEQSAWETAKAKGVDLVVLNPVLVLGPPLQSAINASLVHILKYLTGSAKTYANLTQVYVDVRDVALGHVLVYEAPSASGRYILAETALHRGEVVEILAKFFPEYPLPTKCSDEKNPRAKPYKFTTQKIKDLGLEFKPIKQSLYESVKSLQEKGHLPLPQDSNQNEVIIES

ZmCCR2

MVVFCRKHGQHAFLLEAGDRLVEALEDRHEVHPQILEALVGELVSYHRRVNLVLALGGYGVLREELGEDAAEVAAVQGALGADVAAVRVGRLVHPDVRLGHVPDVHVRLRRVHVGRGRPRQVLHHVVRAGVHRRLQQRAQHQHRVHHHQVQPSLPRHLPRALLRHRLGVAVPVLGVLAILEVAPAGVVHRLAGVTVGVHVHGADGGREHDALHSGVCRRRHHVARAPDRWLDHHLRVIGDRGGGVEDAVTAGEGLGKALWVQQVGAEEDEAVVGAVKGPQVVVLWIHRVPDRAADCVALLQEALDEPGGNEPSSSGHANRPPLPGGQLRRHGRRRLCCWHPS
